# Low-Cost and Lightweight 3D-Printed Split-Ring Resonator for Chemical Sensing Applications

**DOI:** 10.3390/s18093049

**Published:** 2018-09-12

**Authors:** Ahmed Salim, Saptarshi Ghosh, Sungjoon Lim

**Affiliations:** School of Electrical and Electronics Engineering, College of Engineering, Chung-Ang University, 221, Heukseok-Dong, Dongjak-Gu, Seoul 156-756, Korea; ahmedsalim789@gmail.com (A.S.); joysaptarshi@gmail.com (S.G.)

**Keywords:** split-ring resonator, 3D printing, silver-coating, capacitive coupling, microwave sensor

## Abstract

In this paper, a microwave cavity resonator is presented for chemical sensing applications. The proposed resonator is comprised of a three dimensional (3D) split-ring resonator (SRR) residing in an external cavity and capacitively coupled by a pair of coaxial probes. 3D-printing technology with polylactic acid (PLA) filament is used to build the 3D SRR and cavity. Then, the surfaces of the SRR and the inside walls of cavity are silver-coated. The novelty of our proposed structure is its light weight and inexpensive design, owing to the utilization of low density and low-cost PLA. A Teflon tube is passed through the split-gap of the SRR so that it is parallel to the applied electric field. With an empty tube, the resonance frequency of the structure is measured at 2.56 GHz with an insertion loss of 13.6 dB and quality factor (Q) of 75. A frequency shift of 205 MHz with respect to the empty channel was measured when deionized water (DIW) was injected into the tube. Using volume occupied by the structure, the weight of the proposed microwave resonator is estimated as 22.8 g which is significantly lighter than any metallic structure of comparable size.

## 1. Introduction

Additive manufacturing (AM) technology, also called three-dimensional (3D) printing, rapid prototyping, and layered printing, is an approach in which 3D structures are directly printed from a computer-aided design (CAD) file without any part-specific tools and dies [1]. In this freeform fabrication, layers are printed in the horizontal plane and thickness is added by successive repetition. Digital design and CAD tools provide great flexibility to change and modify or control the parameters and process of complex designs anywhere and anytime. Contrary to conventional fabrication techniques (e.g., lithography), in AM the entire structure is completed on a single 3D printer, which also saves production costs and energy. Owing to instant prototyping and the possibility of quick optimization after a single fabrication, academia and researchers have been using 3D printing to test their innovative ideas and concepts. The design and manufacture of lightweight structures are among the most important requirements in the aerospace industry [1]. In this article, 3D printing is utilized mainly to fabricate a lightweight structure, which may offer a low-cost solution without compromising performance.

Fused deposition modeling (FDM) is one of the 3D-printing techniques in which thermoplastic filament is extruded upon heating and objects are built layer by layer using a bottom–top approach. FDM has emerged with printing resolution as high as 50 µm [2]. With the advancement in materials design, low loss polymeric materials such as polypropylene (tan *δ* = 0.000265) have been investigated [3]. Aside the filament material, the internal settings of 3D-printing machine such as build speed, resolution, and heat transfer play their roles in the quality of a printed object.

Metamaterials (MMs) are artificially engineered periodic structures with subwavelength unit-cell dimensions that provide miniaturization and exhibit a high quality factor (Q). The properties of MMs depend mainly on the dimensions, topology, or orientation, and configurations adopted [4]. Split-ring resonators (SRRs) and complementary-split-ring resonators (CSRRs) are the two most used configurations of MMs to accomplish versatile microwave components such as antennas [5], sensors [6], absorbers [7], power dividers [8], and filters [9]. Inductive-capacitive (LC) equivalent circuit models are derived to describe the behavior of unit-cell SRRs, where capacitance is provided by the split-gap of the SRR and inductance is provided by the surface current flowing into the metallic SRR. Single and multiple combinations of planar SRRs fed by various types of transmission lines such as microstrip lines or coplanar waveguides have been widely employed for chemical sensing applications.

There is an increasing trend to manufacture complex designs using 3D-printing technology. For instance, a 3D-printed orthogonal transducer based on modified turnstile junction is presented in Ref. [10]. The multiple bands (Ku-band, lower Ka-band, and upper Ka-band) were targeted. It has been claimed that the 3D metal printing technique has enabled the manufacturing of rectangular waveguide’s corners with a higher accuracy as compared to that of a milling cutter. In Ref. [11], a 3D-printed orthonormal transducer is fabricated using photopolymer resin and after that copper-coating is used. Thus, the structure has an additional feature of being light weight owing to 3D-printing technology. Recently, a few metamaterial structures have also been realized in 3D geometry using various manufacturing technologies.

State-of-the-art 3D resonant structures developed on the basis of SRRs have been proposed [12,13]. In [12], a 3D SRR (18 mm × 18 mm × 3 mm) and a cavity made from metallic materials was proposed as a microwave resonator that can detect chemicals. In [13], 3D SRRs were constructed using a metal stress-driven method. The fabrication strategy consisted of electron-beam lithography and reactive ion etching processes. From the bending test results, it was concluded that the radii of curvature increased with an increase in width while the arm length was fixed. The electric field and magnetic resonance modes both were demonstrated using x-polarized transverse wave. Further, the tunability of these 3D SRRs was investigated in relation to change in temperature. In [14], an SRR probe was proposed that was validated to measure dielectric permittivity of 2 to 27 in the frequency range of 1–3 GHz, with high accuracy and resolution. The tuning capability was demonstrated by changing the geometrical parameters of the SRR. It was proposed to target useful applications such as 2D near-field dielectric imaging of large-area composite materials. In [15], the model of 3D chiral helix-shaped metamaterials was presented. It was shown that the conversion between left and right circular polarization waves for the proposed model was much broader than that of the continuous helix model. The phenomenon of cross-coupling between electric and magnetic fields results from the chiral electric currents on the resonators due to the broken mirror symmetry. For various phase angles, the field distributions on the inner and outer boundary of the rings were analyzed. In [16], a stretchable and bendable SRR was built in a polydimethylsiloxane (PDMS) substrate. This fluidic SRR was realized by injecting liquid metal (Galinstan) into a microfluidic channel, which was realized by combining two PDMS layers. The stress levels onto the surface were transformed into the variation in the Galinstan liquid state. This flexible resonator is a potential candidate for conformal, non-planar, and curved surfaces in flexible electronics.

There are two main reasons behind the use of FDM-based 3D printing technique to fabricate the proposed geometry: (1) Using the 3D printing technique, the geometries (cavity, sensor) have been constructed using lightweight PLA Material and thereafter metal coating has been uniformly deposited. This significantly reduces the overall weight as well as fabrication cost of the design; (2) The cavity has been made using perforated dielectric, to further reduce the amount of the constituent PLA material. This perforated cavity cannot be realized other than with the 3D printing technique. On the contrary, large amount of costly materials are used in [12] to realize sensor structure. In [12], a 3D SRR (18 mm × 18 mm × 3 mm) and a cavity made from metallic materials was proposed as a microwave resonator that can detect chemicals. The choice of materials, such as copper and aluminum, make this design bulky and expensive. In [13], 3D SRRs structures were fabricated using complex fabrication techniques using high-tech machinery. This type of complicated fabrication procedure can be easily ignored in 3D printing techniques, thus reducing the manufacturing time as well as cost. 

In this study, a lightweight and low cost SRR-based chemical sensor has been developed, exploiting 3D printing technology. The cavity perturbation technique has been considered in the proposed geometry for a resulting high Q-factor, which is used to detect various chemicals. Taking an initiative step, a simple design of SRR was chosen to fabricate using 3D-printing technology. Two capacitive coupling probes are used to excite the 3D SRR which is resided in a cavity housing. Both the SRR and the cavity housing are built from Polylactic acid (PLA) material, therefore, structure obtained is lightweight and inexpensive. In order to get conductivity, the surfaces are silver coated. By dint of the cavity housing, radiation losses are reduced. PLA is a low cost and more importantly a very low-density material, therefore, a rigorous weight reduction is expected as compared to any metallic structure of comparable size.

## 2. Sensor Design

**Figure 1:**
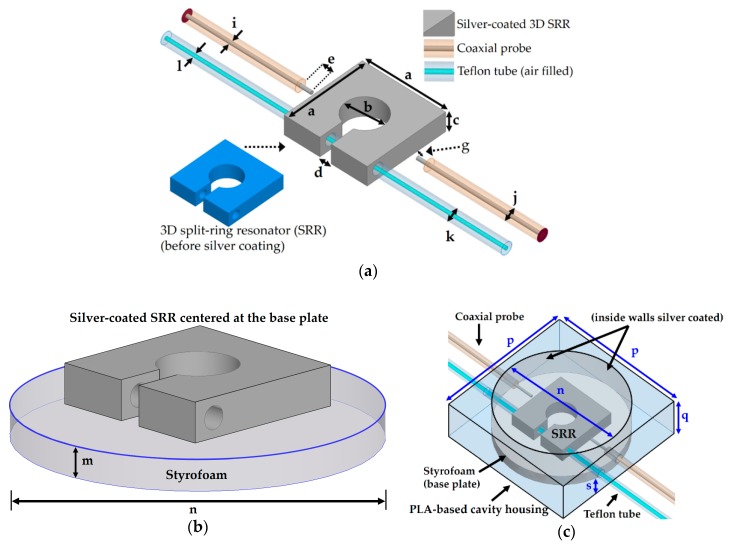
Design parameters of the final layout of the proposed microwave chemical sensor: (**a**) Silver-coated 3D split-ring resonator (SRR) and capacitively coupled coaxial probes, (**b**) SRR centered on the base plate (Styrofoam), and (**c**) silver-coated 3D SRR residing in a cavity housing and capacitively coupled through two coaxial cables.

The working principle of the proposed microwave resonator is based on cavity perturbation phenomenon. A 3D SRR is placed inside a cavity and a Teflon tube is passed through the SRR so that the tube is parallel to the electric field of the incident EM wave. The SRR along with the air-filled tube results in a response, obtained from the transmission coefficient (S_21_) plot. When a target chemical is injected into the tube, the response shifts due to the change in the effective permittivity value, and the chemical can be easily detected from that shift in resonance. The final layout of our proposed microwave resonator is shown in Figure 1; we describe the detailed design process herein.

**Figure 2:**
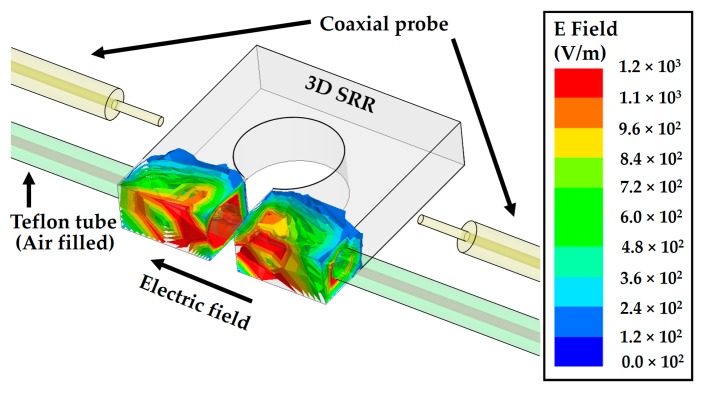
Electric field magnitude distribution at a resonance frequency of 2.56 GHz with an empty Teflon tube passing through a silver-coated 3D SRR resided in a cavity, which is capacitively coupled by a pair of coaxial probes. The high electric field in the proximity of the SRR split gap is ascribed to the high capacitance in the gap. A high frequency structural simulator (HFSS) is used for simulation analysis.

In our study, the 3D SRR consisted of PLA material, which can be made electroconductive when finite conductivity of silver is assigned in the simulations. To consider the fine finish of the 3D-printed sample and to observe the resonant frequency around 2.5 GHz, the 3D SRR with a size of 22 mm × 22 mm × 5 mm (*a* × *a* × *c*) was considered. The split gap of the SRR was *d* = 3 mm wide and the diameter of the center hole was *b* = 10 mm. The highest electric field region was located around the split gap of the SRR, as is obvious owing to higher capacitance that exists in the split gap. Taking into accounts the effects of the microfluidic tube, an air-filled Teflon tube was passed through the high electric field region of the SRR; the resultant E field magnitude distribution is shown in Figure 2. The liquid-filled tube parallel to the E field gives the device higher sensitivity compared to if the same tube is considered perpendicular to E field [12]. The Teflon tube specifications were set as outer diameter *k* = 3 mm and the inner diameter *l* = 1.26 mm, which were chosen by considering the thickness (*c*) of the 3D SRR. 

**Figure 3:**
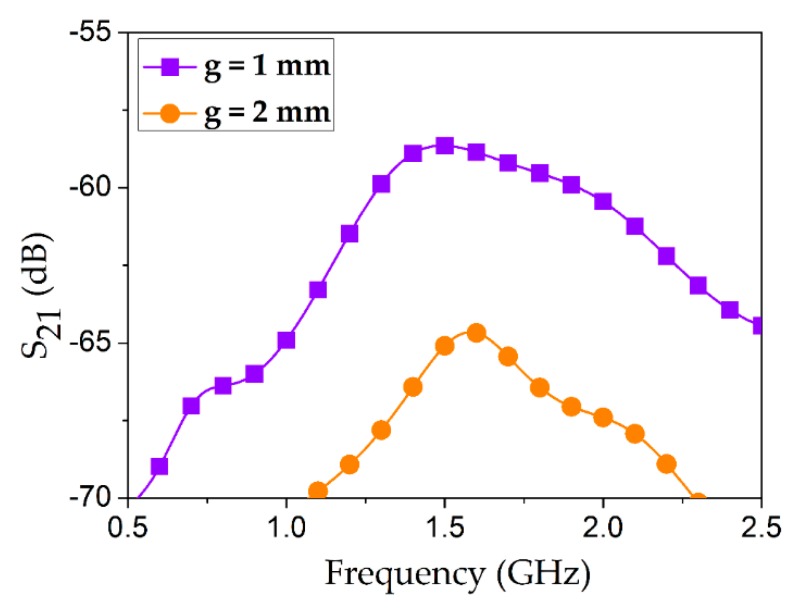
Insertion loss (magnitude of S_21_) profile when a silver-coated 3D-printed SRR is excited using two capacitively coupled coaxial probes. Inset shows the complete assembly considered for this set of simulations. The coupling gap (*g*) was varied and the corresponding insertion loss values are shown. The curves are not sharp, and minimum IL is 58 dB, which is too lossy to consider for practical scenarios. This necessitates an additional assembly or modification in the design to reduce the losses and to obtain sharp resonances.

A pair of 50-ohm RG316 coaxial cables were considered, which provide low attenuation (approximately 50 dB per 100 feet @ 3 GHz). Despite the low loss profile of the coaxial probes, considerably high IL was observed in simulation when they were capacitively coupled with the silver-coated 3D SRR, as shown in Figure 3. The conductivity of silver coating was set as *σ* = 10^6^ S/m. In order to design the 50-ohm criterion of the RG316 coaxial probe, the specifications were taken from the data sheet such that the diameter of the inner conductor was *i* = 0.9 mm and the diameter of the outer conductor was *j* = 3 mm. Insertion loss (IL) values in microwave engineering are considered to be positive, as it indicates the loss incurred due to insertion of a particular geometry in a device. This value is generally equal to the negative of the transmission coefficients (S_21_). Since S_21_ is turned out to be in negative domain, IL will be in the positive scale. Minimum IL values were 58 dB and 64 dB when coupling gaps were *g* = 1 mm and *g* = 2 mm, respectively. Very high IL values suggest that the pertinent assembly and design are infeasible in practical scenarios. This is the reason we opted for a cavity housing platform to surround the silver-coated 3D SRR. The 3D SRR resides in the cavity housing, and its resonance frequency is changed upon injection/removal of the target chemicals [12]. The cavity perturbation technique is widely used in the microwave resonant structures that have been employed for chemical sensing applications [17,18,19].

A square-shaped box with a hollow center part was envisaged as the cavity housing, and the 3D SRR is placed in the cavity, as shown in Figure 1. Two coaxial probes were used to transfer energy in an efficient way so that the SRR could be excited, and the cavity perturbation phenomenon was observed. The distance between the inner conductor of the coaxial probe and the SRR is defined as the coupling gap (*g*). The inner sides of the cavity were assigned with the conductivity of silver (*σ* = 10^6^ S/m). A circular-shaped plate made of Styrofoam was placed on the silver-coated base of the cavity housing, as shown in Figure 1b. Its height and diameter were *m* = 4 mm and *n* = 44 mm, respectively. The silver-coated 3D SRR was centered on the Styrofoam base plate. In simulations, two different materials (PLA and Styrofoam) were investigated as the base plate for the SRR geometry, however, Styrofoam was chosen in the final design due to its low loss characteristic (see Figure 1b). The dielectric properties of the PLA material (*ε_r_* = 2.48 and tan *δ* = 0.02) [20], Teflon tube (*ε_r_* = 2.1 and tan *δ* = 0.0011), and Styrofoam (*ε_r_* = 1.001 and tan *δ* = 0.0002) were used in this study. The dimensions of the square-shaped cavity housing were set as follows: side length *p* = 50 mm, height *q* = 15 mm. The Styrofoam (circular plate) resided on a 1-mm-thick PLA base, which is represented by s (see Figure 1c).

**Figure 4:**
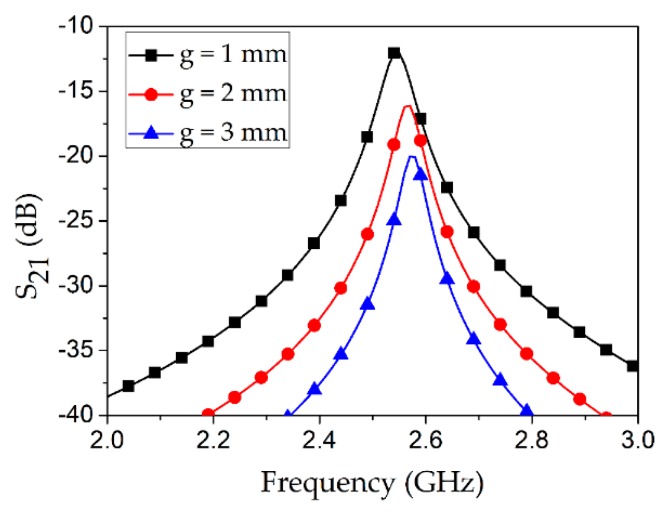
Coupling gap (*g*) investigation for the proposed microwave cavity resonator when the Teflon tube was empty.

As shown in Figure 4, a parametric analysis of the coupling gap (*g*) with an air-filled Teflon tube was conducted. Among three cases, the minimum insertion loss of 12 dB was obtained at *g* = 1 mm which is expected because the energy transfer increases as the coupling gap decreases.

**Figure 5:**
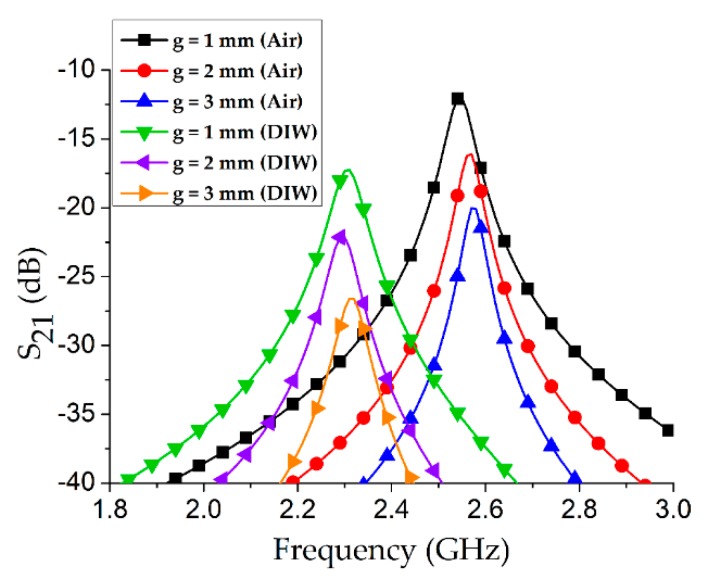
Investigation of optimum coupling gap (*g*) based on the criterion of maximum frequency shift (Δ*f*) corresponding to dielectric properties of air and deionized water (DIW). The optimum coupling gap is found as *g* = 2 mm because at this coupling the higher frequency shift (Δ*f* = 270 MHz) as compared to other two cases of *g* = 1 mm and 3 mm, is achieved.

Although the coupling gap (*g*) was initially optimized as 1 mm, the Teflon tube was kept empty during this investigation. However, the target application of our proposed microwave cavity resonator is chemical sensing, and therefore we re-investigated the coupling gap (*g*) with the Teflon tube filled with some particular solvent/chemical. An individual frequency shift with an empty channel vs. a water-filled tube was estimated for several values of *g*, as shown in Figure 5. A maximum frequency shift of 270 MHz was obtained during the coupling gap (*g*) of 2 mm, and hence this value was considered as the final value in our proposed sensor structure.

**Figure 6:**
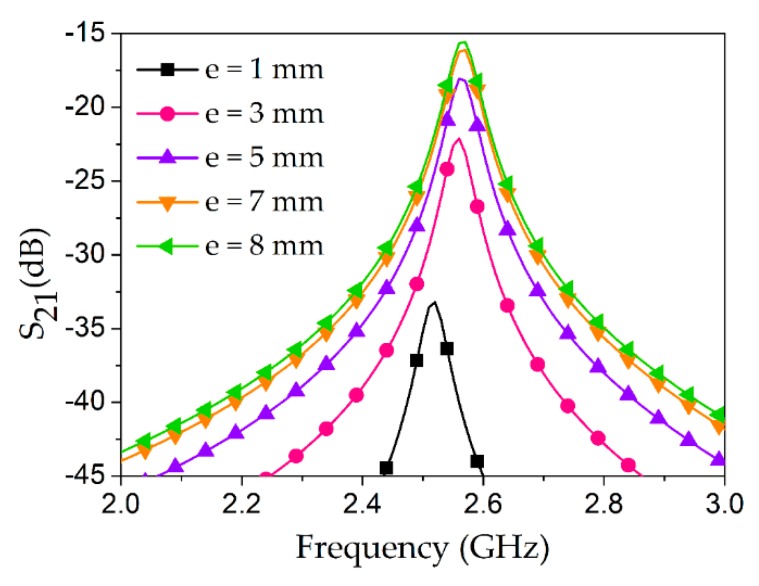
Various lengths of bare inner conductor (*e*) of the coaxial coupling probe were investigated, where the coupling gap was fixed as *g* = 2 mm in this set of simulations. Inset shows the top view of the assembly for the case when *e* = 7 mm. The bare inner conductor of the coaxial cable is not radiating as a monopole antenna and it does not contribute to the high transmission coefficient (or reduced losses), thus ensuring that the length of the coaxial cable is less than one wavelength (*λ*), and the length of the bare inner conductor is less than *λ*/4.

Minimizing the IL is necessary to maintain a high Q-factor, which directly influences the sensitivity of the proposed sensor. In addition to the coupling gap, the length of the bare inner conductor (peeled-off part represented by *e*) is an important consideration. In order to find the optimized *e*, a parametric analysis was conducted while keeping *g* = 2 mm fixed during this set of simulations (see Figure 6). As shown in the figure, *e* = 1 mm and 3 mm cause high IL values, such as 33 dB and 22 dB, respectively, whereas *e* = 7 mm and 8 mm provide reasonable values of IL, such as 16 dB and 15.5 dB, respectively. Therefore, *e* = 7 mm was considered for the final layout of our proposed design.

The resonance frequency (*f*_r_) of our proposed design mainly depends on the physical dimensions of the SRR geometry and the chemical under test. The other dimensions (for instance length of the inner conductor *e* and coupling gap *g*) are loosely influencing the resonance, through mutual coupling effect. Instead, these parameters (*g* and *e*) are mainly contributing to the magnitude of S_21_ as depicted in Figure 3, Figure 4, Figure 5 and Figure 6. Hence the shift is not significant with different values of *g*, as observed in Figure 5. Nevertheless, we have considered *g* = 2 mm as the best coupling gap with the water-filled Teflon tube.

All design parameters of the final layout of our proposed microwave chemical sensor are provided in Table 1.

## 3. Simulation Analysis

**Figure 7:**
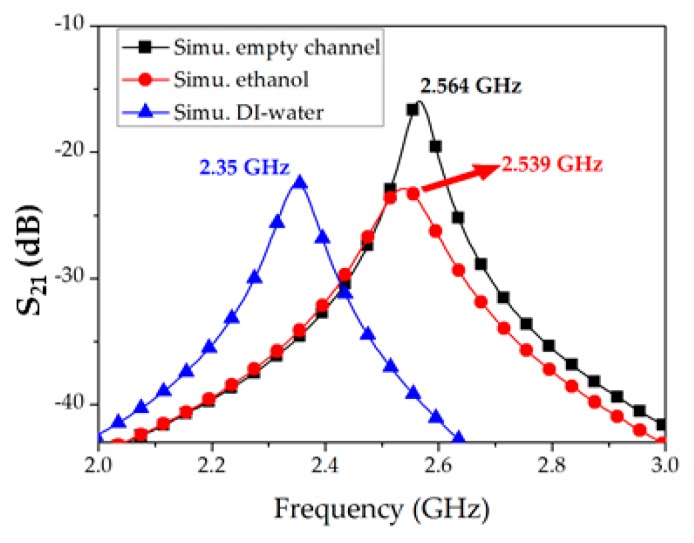
Simulation analysis of our proposed microwave resonator working as a chemical sensor. S_21_ corresponding to empty channel, ethanol, and deionized water (DIW) are shown.

ANSYS High-frequency structure simulation (HFSS) was used for full-wave simulations of our proposed idea; S-parameters were obtained after inserting the dielectric properties of air, ethanol, and deionized water (DIW). The dielectric properties of ethanol (*ε_r_* = 6.5 and tan *δ* = 0.96) and DIW (*ε_r_* = 76 and tan *δ* = 0.12) were adopted from a standard reference [21], and the S_21_ curves of our proposed sensor were analyzed (see Figure 7). The resonance frequency with an empty channel was found at 2.564 GHz with an IL of 15.93 dB. The resonance frequency and IL with ethanol were found at 2.539 GHz and 22.87 dB. With respect to an empty channel, a frequency shift of 25 MHz when the dielectric properties of ethanol were simulated is attributed to the difference in the dielectric constant between air and ethanol (5.5), which is a low number. When the dielectric properties of DIW were simulated, the resonance frequency and IL were 2.35 GHz and 22.44 dB. A maximum 214-MHz shift in resonance frequency (simulations) corresponding to the empty channel and DIW was observed.

## 4. Fabrication

**Figure 8:**
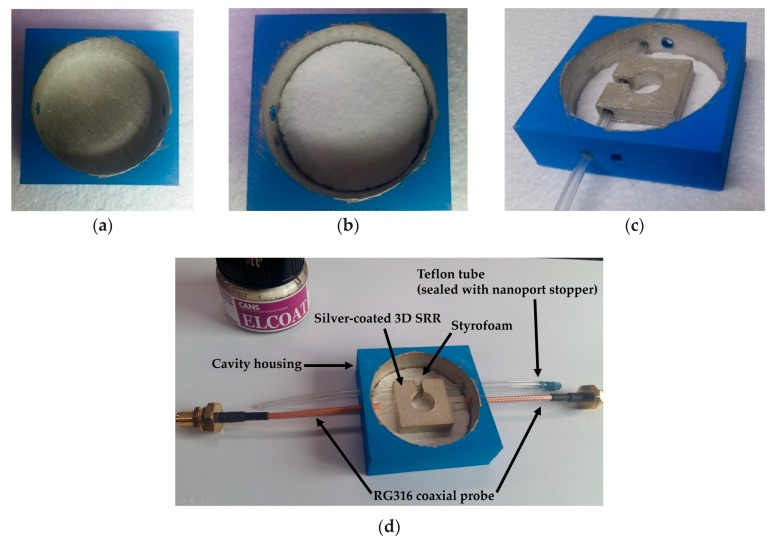
Stepwise fabrication of the silver-coated 3D-printed SRR residing in a cavity housing and capacitively coupled through two coaxial cables (RG316): (**a**) Cavity housing with silver-coated inside walls and base, (**b**) Styrofoam as a base plate in the cavity housing, (**c**) silver-coated SRR along with a Teflon tube passing through it and centered on the Styrofoam, and (**d**) complete assembly of our proposed structure ready for measurements.

In order to fabricate our proposed microwave cavity resonator, digital files (geometry specified) are provided to 3D printer Ultimaker 2+ and PLA filament is used as the constituent material. After fabrication of the 3D designs, the inside wall of the cavity housing and the surfaces of the 3D SRR were silver-coated (CANS ELCOAT, P-100) using a paint brush. For drying purposes, the silver-coated sample was kept for 24 h at room temperature. A circular Styrofoam piece (4 mm × 44 mm) was cut and placed inside the cavity housing. The silver-coated SRR with a Teflon tube passing through it was placed at the center of the Styrofoam. A pair of 50-ohm coaxial cables (RG316) was capacitively coupled to the SRR at a distance of 2 mm, which was roughly adjusted with a 2-mm thick plastic film that served as a spacer. Stepwise fabrication of the 3D cavity housing and SRR is shown in Figure 8a–c; Figure 8d shows the complete structure of our proposed resonator working as a chemical sensor.

In the proposed sensor, metal coating (silver paste) has been deposited uniformly on the PLA material-based sensor and the cavity. The thickness of the silver paste has been maintained such that it is larger than the skin depth (9.95 µm at 2.56 GHz for silver metal) and hence no current reaches the PLA material. Thus, the use of PLA in our design is to make the structure lightweight and inexpensive only. Because the loss property of the PLA has not been utilized in the structure, we have chosen the best fitted material in the 3D printer irrespective of its lossy properties. Due to manual painting, the thickness of the silver paste might be non-uniform at some points, which can be addressed in the future works.

## 5. Measurements

**Figure 9:**
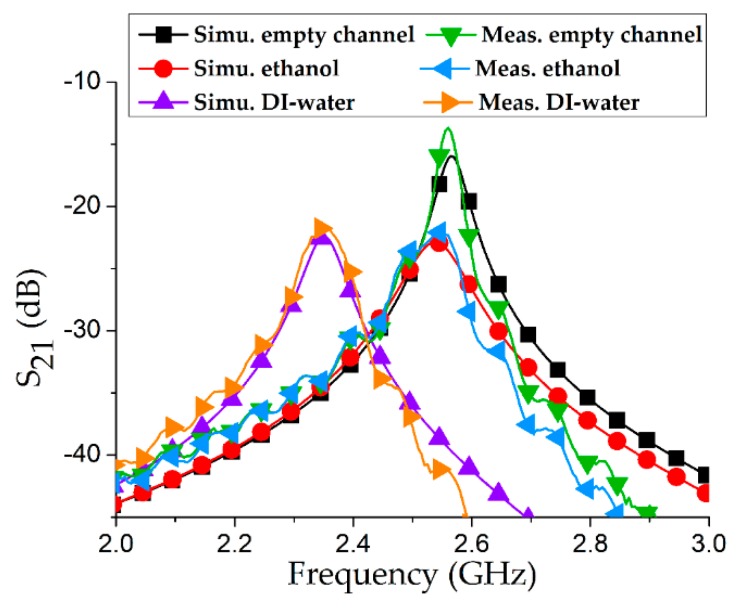
Simulations and measurements of insertion loss (magnitude of S_21_) obtained from our proposed microwave resonator when an empty channel, ethanol, and DIW were analyzed.

Coaxial probes were connected to a VNA (MS2038C, Anritsu Corporation, Atsugi shi, Japan) and frequency response was recorded from 1–3 GHz after a full two-port short, open, load, thru (SOLT) calibration. The transmission coefficient (S_21_) was measured at 2.56 GHz with an empty channel, and the IL (magnitude of S_21_) was 13.66 dB. To verify the exactness of the measurements, simulation and measurement results with an empty channel were drawn together and found to have excellent agreement. To proceed further, 100% ethanol and DIW were individually injected into the Teflon tube and in each case the resonance frequency (*f_r_*) and S_21_ were measured. Simulations and measurements of the three considered cases (empty channel, ethanol, DIW) are shown in Figure 9.

To validate our proposed idea of a 3D-printed silver-coated SRR as a chemical sensor, the resonance frequency, Q factor, and IL from both simulations and measurements are provided in Table 2. 3 dB bandwidth of a microwave device indicates the bandwidth around the center frequency, where the response gets reduced by 3 dB with respect to the response at its peak. Q factors are estimated from S_21_ curves (simulations and measurements) based on 3 dB bandwidth criterion such that Q = *f_r_*/Δ*f*_3dB_ [22,23]. Relative error in resonance frequency was estimated from the difference between the corresponding simulations and measurements [24,25].

When we measure the resonant frequency of liquids, we measured it three times. For instance, the resonant frequencies of ethanol are measured to be 2.544 GHz, 2.547 GHz, and 2.543 GHz for the first, second, and third measurement, respectively. Therefore, the average frequency of three measured frequencies is 2.545 GHz. The frequency variation of each frequency is 1 MHz, 2 MHz, and 2 MHz for each time. Because the resonant shift of 25 MHz is much larger than this frequency variation of 2 MHz, we can detect the frequency shift from air to ethanol or DIW.

## 6. Discussion

The main attraction in our proposed resonator is its lightweight and inexpensive design owing to utilization of 3D-printing technology. In our proposed microwave resonator, PLA and Styrofoam materials were used. Because the density of PLA (1250 kg/m^3^) and Styrofoam (30 kg/m^3^) are much lower than the density of copper (8930 kg/m^3^) and aluminum (2700 kg/m^3^), the structure of our proposed microwave resonator is significantly lighter compared to that of [12]. From the dimensions provided in Table 1, net volume occupied by the proposed structure is estimated and compared with the 3D SRR presented in reference [12]. Our proposed 3D-printed SRR and cavity achieved, 61% and 85% weight reduction, respectively, as compared to the 3D SRR and cavity utilized in [12].

In order to evaluate the performance of our proposed microwave chemical sensor, a comparison with existing devices is provided in Table 3. Sensitivity (S) was estimated as S (%) = Δ*f*_max_/*f*_r_, where Δ*f*_max_ is the maximum frequency shift when DIW was injected, and *f*_r_ is the resonance frequency. The sensitivity (S) of our proposed microwave chemical sensor is comparable to the recently proposed microwave chemical sensors.

In addition, 3D printing, as an additive manufacturing technology, adds valuable features such as an etching-free and chemical-less processing. In [24] and [27], the structures were realized using photolithography, which is a subtractive manufacturing process. It requires various steps and a particularly clean environment and involves hazardous chemicals. In [26], the design complexity is increased due to fabrication of five stacked layers. Our proposed microwave cavity resonator can be a potential candidate to be used for chemical sensing applications. Thus, the overall weight of the proposed assembly is almost six times lighter as compared to the previously reported 3D SRR and cavity. Although the overall volume is constant, the structure is very lightweight, low cost, and practically useful. 

The material cost is not standardized across the world and hence cannot be estimated. However, a general statement can be said based on the experience and universal price. For instance, copper and aluminum (materials used in [12]) are much costlier than PLA and Styrofoam (materials used in our study).

## 7. Conclusions

In this paper, a 3D-printed SRR residing in a cavity is presented as a chemical sensor. The novelty of the proposed structure is its light weight and low cost. 3D-printing technology has additional attractive advantages that are not found in photolithography. The SRR and cavity both were printed using PLA material and silver-coating was used to make the surfaces electroconductive. 3D SRR was centered on a Styrofoam base plate and both were placed in the cavity housing. A pair of coaxial cables was capacitively coupled to the SRR and the structure resonated at 2.56 GHz with an empty tube passing through the SRR. To validate our proposed idea, the transmission coefficients (S_21_) were measured with ethanol and DIW. A frequency shift of 205 MHz with respect to the empty channel was achieved when DIW was injected into the channel. 3D-printing technology can play an important role in microwave designs, which are bulky or complex to fabricate using photolithography.

## Figures and Tables

**Table 1:** Design parameters of our proposed 3D-printed SRR and its outer assembly, which are proposed as a chemical sensor. (Units: mm).

Parameter	Value	Parameter	Value	Parameter	Value	Parameter	Value
a	22	e	7	k	3	p	50
b	10	g	2	l	1.26	q	15
c	5	i	0.9	m	4	s	1
d	3	j	3	n	44		

**Table 2:** Comparison of simulations and measurements. The resonant frequency (*f_r_*), Q-factor, and insertion loss (IL) for the three cases (air, ethanol, and DIW) are presented.

	Simulations *f_r_* (GHz)	Measurements *f_r_* (GHz)	Simulations Q	Measurements Q	Simulations IL (dB)	Measurements IL (dB)	Relative Error in *f_r_* (%)
Air	2.564	2.559	51.28	75.82	15.93	13.66	0.195
Ethanol	2.539	2.545	28.21	24.71	22.87	22.09	0.236
DIW	2.350	2.350	35.1	25.54	22.44	22.62	0

**Table 3:** Performance comparison of recently published microwave chemical sensors utilizing various manufacturing technologies.

Reference	Technology	Manufacturing	Weight (g)	*f_r_* (GHz)	Δ*f*_max_ (MHz)	S (%)
This work	3D SRR	3D-printing	22.8	2.56	205	8
[12]	3D SRR	Milling machine	140.1	2.53	60	2.37
[24] ^†^	SIW *	Photolithography	N/A	5.81	470	8
[26] ^†^	Dual mode resonator	LTCC **	N/A	3	300	10
[27] ^†^	Planar SRR	Photolithography	N/A	0.87	100	11.49

SIW represents * substrate-integrated waveguide. LTCC ** represents low-temperature co-fired ceramics. N/A represents not available. ^†^ They are consisted of planar structures and metallic patterns are fabricated on conventional circuit board.
